# Post-Vaccination Sero-Monitoring of Peste des Petits Ruminants in Sheep and Goats in Karnataka: Progress towards PPR Eradication in India

**DOI:** 10.3390/v16030333

**Published:** 2024-02-22

**Authors:** Vinayagamurthy Balamurugan, Rakshit Ojha, Kirubakaran Vinod Kumar, Anand Asha, Suhail Ashraf, Annett Helcita Dsouza, Archana Pal, Prajakta Prashant Bokade, Shakuntala Krishnaiah Harshitha, Ramchandra Deshpande, Mahadevappa Swathi, Kuralayanapalya Puttahonnappa Suresh, GurrappaNaidu Govindaraj, Subramanya Prasad Hasnadka, Shanmugam ChandraSekar, Divakar Hemadri, Anirban Guha, Njeumi Felix, Satya Parida, Baldev Raj Gulati

**Affiliations:** 1 Indian Council of Agricultural Research, National Institute of Veterinary Epidemiology and Disease Informatics (ICAR-NIVEDI), Yelahanka, Bengaluru 560064, India; ricky.ojha@gmail.com (R.O.); vinodkmr33@gmail.com (K.V.K.); ashareddy457@gmail.com (A.A.); suhailashraf9906@gmail.com (S.A.); annettdsouza4099@gmail.com (A.H.D.); archanapal307@gmail.com (A.P.); prajb95@gmail.com (P.P.B.); harshi79sk@gmail.com (S.K.H.); dramchandra643@gmail.com (R.D.); swathi.par10@gmail.com (M.S.); suresh.kp@icar.gov.in (K.P.S.); govindaraj.naidu@icar.gov.in (G.G.); divakar.hemadri@icar.gov.in (D.H.); baldev.gulati@icar.gov.in (B.R.G.); 2 Commissionerate of Animal Husbandry and Veterinary Services, Pashupalana Bhavana, Hebbal, Bengaluru 560024, India; ddpddlahvs@gmail.com; 3 Indian Council of Agricultural Research, Indian Veterinary Research Institute (IVRI), Mukteswar, Nainital 263138, India; schand_vet@yahoo.co.in; 4 Department of Animal Husbandry & Dairying, Krishi Bhawan, New Delhi 110001, India; anirban.guha@gov.in; 5 Food and Agriculture Organization of the United Nations (FAO), Viale delle Terme di Caracalla, 00153 Rome, Italy; felix.njeumi@fao.org (N.F.); satya.parida@fao.org (S.P.)

**Keywords:** PPR eradication, GCES guidelines, mass vaccination, post-vaccination sero-monitoring, vaccine efficacy, epidemiological unit, Karnataka state, India

## Abstract

Peste des petits ruminants (PPR) presents economic challenges in enzootic countries impacting small ruminant productivity. The state of Karnataka, India, implemented a mass vaccination campaign in alignment with the PPR-Global Eradication Programme (GEP) and the National Strategic Plan for PPR eradication. This study was conducted from January to March 2023 to assess seroconversion in post-vaccinated goats and sheep at the epidemiological unit (epi-unit) level, aligning with the World Organisation for Animal Health (WOAH) and the Food and Agriculture Organization (FAO) guidelines in the PPR Global Control and Eradication Strategy (GCES). Before vaccination, 3466 random serum samples were collected from small ruminants of three age groups (6–12 months, 1–2 years, and >2 years) across 116 epi-units, spanning 82 taluks in 28 districts. Post-vaccination sero-monitoring included 1102 serum samples collected from small ruminants of the 6–12-month age group only, across 111 epi-units covering 64 taluks in 23 districts. The PPRV antibody status was determined using an indigenous hemagglutinin (H) protein monoclonal antibody-based competitive ELISA kit. Pre-vaccination, the PPR seropositivity rates were 55%, 62%, and 66% in the age groups of 6–12 months, 1–2 years, and >2 years, respectively, with a 61% PPRV antibody prevalence across all the age groups. Notably, 41% of the epi-units exhibited antibody prevalence rates of ≥70%, indicating a substantial population immunity, possibly attributed to the previous vaccination program in the state since 2011. In contrast, only 17% of the epi-units had below 30% seroprevalence rates, emphasizing the need for intensified vaccination. Statistical analysis of the data revealed significant correlations (*p* < 0.05) between the presence of PPRV antibodies and host factors such as species, breed, and sex. Post-vaccination seroprevalence in the 6–12 months age group was found to be 73.4%, indicating the use of an efficacious vaccine. On the evaluation of vaccination immunity in the 6–12 months age group, it was revealed that over 69% of the epi-units achieved a response surpassing ≥70%, indicating a significant improvement from 42% of the epi-units in pre-vaccination. For active PPR eradication, a mass vaccination campaign (>95% coverage) targeting small ruminant populations aged >4 months is advocated, aiming to achieve the desired herd immunity of >80%. This study offers crucial insights into PPR baseline seroprevalence/immunity status and vaccine efficacy, guiding national strategies towards a PPR-free India and further supporting the global eradication initiative.

## 1. Introduction

Peste des petits ruminants (PPR), commonly known as ‘Small ruminants plague’, is a highly contagious viral disease that primarily affects both domestic and wild small ruminants. The World Organisation for Animal Health (WOAH) recognizes PPR as a notifiable transboundary animal disease. PPR is caused by the PPR virus (PPRV), a member of the *Morbillivirus* genus in the *Paramyxoviridae* family. Clinical manifestations in goats and sheep include elevated temperature, oculo-nasal discharges, ulcers, gastroenteritis, and bronchopneumonia [1]. The economic implications of PPR are profound, especially in endemic regions spanning the Arabian Peninsula, the Middle East, and major parts of sub-Saharan Africa and Asia [1,2,3]. Global initiatives, including the PPR Global Eradication Programme (PPR-GEP) and the PPR Global Control and Eradication Strategy (GCES), have been launched to combat PPR [4]. The Food and Agriculture Organization (FAO) has initiated the second phase of the PPR-GEP and unveiled a blueprint for recommended activities covering 2022–2030, aiming to achieve global PPR eradication by 2030 [5].

In India, PPR has remained endemic since 1994 in goats and sheep, with multiple outbreaks reported every year [1,2]. This has prompted measures such as vaccination, disease reporting, rapid diagnosis, and surveillance [1]. Temporal and spatial epidemiological analysis of national surveillance data has revealed PPR as a leading cause of mortality in goats and sheep, accounting for 36% of deaths [1]. India’s annual financial loss due to PPR is estimated at INR 16,110 million (USD ~200 million) [3]. Even before the global PPR-GEP framework was established in 2017, India initiated a national control program for PPR (PPR-CP) in 2011 [1,6]. The core components of PPR-CP encompass vaccination, rapid diagnosis, surveillance, and post-vaccination sero-monitoring for disease prevention [7]. India’s vaccination response to PPR began in 2006 with focal vaccination in outbreak places, with mass vaccination disease control campaigns initiated in the Southern Peninsular regions in 2011, followed by mass vaccination across the entire country since 2014 [1,6,8]. The Department of Animal Husbandry and Dairying (DAHD), Government of India (GoI), has further expanded the countrywide mass vaccination (covering all susceptible domestic small ruminant populations above four months of age to achieve an immunity level of >70%) by launching the PPR Eradication Programme (PPR-EP). The government is working towards PPR eradication by 2030 under the National Strategic Plan (NSP) for PPR Eradication.

Despite vaccination efforts in India, PPR outbreaks are still reported in parts of the country, mainly due to bottlenecks in countrywide surveillance [1,9,10]. Prevalence studies to assess PPRV antibodies and immunity status are crucial for PPR eradication, including post-vaccination evaluation (PVE) or sero-monitoring to determine vaccine efficacy and vaccination effectiveness. The present study was conducted in Karnataka, one out of the 30 states of India, from January to March 2023 to establish the baseline prevalence and immunity status of PPR in sheep and goats before the implementation of mass vaccination during 2023 under PPR-EP (eradication program). Additionally, the study seeks to evaluate the post-vaccination immune response (sero-monitoring) and assess overall vaccination effectiveness. 

## 2. Materials and Methods

### 2.1. Study Area

**Figure 1:**
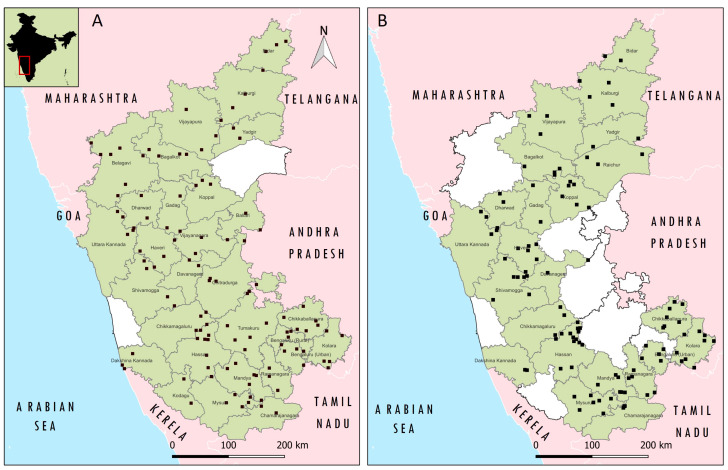
The location of surveyed epidemiological units (villages) for collection of serum samples are depicted (as filled square) in the GIS Map of the Karnataka state, India. (**A**) Survey for prevalence/immunity status of PPR before implementation of mass vaccination, Red box in India map indicated the location of the studied state (**B**) Survey for post-vaccination sero-monitoring of PPR.

Karnataka state was selected for this study because of its early initiation of the PPR-CP in 2011, resulting in a significant decrease in PPR outbreaks [1,6]. Moreover, it is the first state to implement mass vaccination under the NSP for PPR Eradication in 2023. Karnataka, the sixth-largest state in India, comprises 30 districts covering 191,791 square kilometers (5.83% of India’s total area). Unique in its geography, Karnataka is the only southern state bordering with all its southern counterparts (Figure 1). It is surrounded by the Arabian Sea to the west and shares borders with six other states: Goa, Maharashtra, Telangana, Andhra Pradesh, Tamil Nadu, and Kerala [6]. According to the 20th livestock census of India in 2019, the sheep and goat population in Karnataka was 17.21 million, with sheep accounting for 11.05 million (http://www.dahd.nic.in accessed on 5 January 2023).

### 2.2. Study and Target Population

The study population comprised all susceptible sheep and goats in Karnataka. A village was considered as an epidemiological unit (epi-unit) due to its distinct socio-economic and animal husbandry practices. Target animals within these epi-units were categorized into three age groups, i.e., 6–12 months, 1–2 years, and >2 years.

### 2.3. Sampling Design

The sampling design, sample size estimation, and sampling methods for the post-vaccination sero-monitoring of PPR in sheep and goats were carried out as per the described Post Vaccination Evaluation (PVE) protocol of the WOAH/FAO guidelines [4,7]. Briefly, a cross-sectional study was undertaken to determine the seroprevalence of antibodies against PPR in sheep and goat populations before the implementation of mass vaccination under the PPR-EP in 2023, utilizing a stratified sampling plan under the strategic PVE protocol I approach of the WOAH/FAO guidelines [7]. The random sampling plan for small ruminants was devised in epi-units across different districts in Karnataka. A two-stage random sampling procedure was implemented with a fixed level or the required number of cluster units/epi-units designated for PVE. The convenient allocation was utilized to collect serum samples from randomly selected households/flocks involved in sheep and goat rearing. A list of villages (epi-units) in various districts of the state and their respective sheep and goat populations was compiled. Randomization of epi-units was carried out, selecting those with a substantial population (more than 500 heads) as inclusion criteria, using the National Institute of Veterinary Epidemiology and Disease Informatics (NIVEDI’s) epi-calculator software (https://www.nivedi.res.in/Nadres_v2/Epical/stratified/random_sampling.php accessed on 5 January 2023), as described previously [8]. The sampling method ensured the representation of three to four households or flocks (each with a minimum of 20 animals) to be selected in each of the chosen epi-units.

### 2.4. Sample Size Estimation

The GCES guidelines of WOAH/FAO [4,7] was followed for sample size estimation. In the proposed sample sizes for the selected protocol I adhered to the principles outlined in the WOAH/FAO guidelines. They were adjusted to reflect the current epidemiological conditions in the country. In brief, to calculate the sample size of the epi-units with a specified level of confidence intervals (CI) and precision, assuming an unknown large population of epi-units, the formula *n* = (Z^2^ × P (1 − P))/e^2^ was used. Here, Z represents the value from the normal distribution, P is the expected proportion of epi-units protected, and e is the desired precision. Where n is the sample size for the state, Z is the 95% confidence level (standard normal value of 1.96), p is the prevalence, which was to be taken as 30% for pre-vaccination and 70% was taken as the immune population at field level for post-vaccination sero-monitoring, as per the GCES guidelines. ‘e’ is the precision of the sample size estimate and is normally set at 10% (0.10), as is the acceptable sampling error. Additionally, to account for the variation in the sensitivity/specificity of the diagnostic assay to be employed [8], the sample size estimation considered inputs such as unit-level design prevalence (30%), cluster-level design prevalence (3%), target cluster sensitivity (92.4%), and specificity (98.4%) of the assay (PPR c-ELISA kit) [8]. The target system sensitivity was set at 95% confidence interval. Based on these inputs, the total epi-units/clusters sample size was estimated to be 108–120 epi-units in Karnataka.

In each of the selected epi-units, the number of secondary units (animal unit samples) was calculated using the hypergeometric distribution as per the GCES guidelines, considering an animal unit-level prevalence of 30% in small ruminants [4,7]. Since the total number of sheep and goats in the selected epi-unit was over 94, the animal unit samples of 9 or 10 were to be collected, based on the minimum sample size required to assess the seroprevalence and sero-monitoring of PPR at a 95% confidence interval. Thus, a minimum of 3240 secondary animal unit samples (108 epi-units × 30 animal units) from all three age groups was collected to study pre-vaccination immune status/study before the implementation of mass vaccination. In contrast, the PVE sero-survey was carried out using the above sampling design after 60 days but before 90 days post-vaccination. The minimum number of days between vaccination and post-vaccination sampling, as well as the minimum period required to detect antibody levels (14–21 days), was ensured by employing the above sampling design. The post-vaccination sero-monitoring plan targeted the 6–12 months age group only, assuming they had fewer chances of contact with the vaccine or the virus before the day 0 vaccination campaign as per the WOAH guidelines. Further, it was assumed that at least 50% of the vaccinated epi-units would have at least 70% of its population seropositive. A maximum of 9–10 samples were collected from the 6–12-months old animals only, from three or four selected flocks in each of the randomly selected epi-units. Thus, for the PVE serosurvey, a minimum of 1080 secondary animal unit samples (108 epi-units × 10 animal units) were collected for post-vaccination sero-monitoring (Table S1).

### 2.5. Samples and Data Collection

During the study period from January to March 2023, in each epi-unit, in the first survey (pre-vaccination survey before the start of mass vaccination) serum samples were collected from 30 animal units (sheep and goats), with a maximum of 10 samples for each of the three different age groups. A maximum of only 3–4 animals from each eligible age group (between 6–12 months, 1 to 2 years, and >2 years or between 6–12 months = 4, >one year = 6) were conveniently collected in each randomly selected flock/household. Whereas, in the second survey (post-vaccination survey) 10 samples from the 6–12 months age group only were collected for post-vaccination sero-monitoring [7]. The state conducted mass vaccination campaigns in January 2023, covering more than 23 districts in the first month and subsequently extending coverage to the remaining districts. For both pre- and post-vaccination sampling, in the epi-unit where only either sheep or goats were present, a maximum of animal samples of either species were collected. This sampling was conducted with the involvement of the Karnataka State Animal Husbandry and Veterinary Service Department; additionally, a survey was conducted using a questionnaire. The surveyed villages before vaccination (*n* = 116) and post-vaccination (*n* = 111) are shown in the GIS Map (Figure 1A,B) based on their geographical coordinates, utilizing QGIS Software 2.18.6 version. The survey questionnaire was used to collect data relevant to host factors (species, age, breed, sex, etc.) to ascertain possible animal-level host and epidemiological factors. The samples were transported to the WOAH Reference Laboratory Network for PPR-South India (ISO/IEC 17025:2017 Accredited) at ICAR-NIVEDI, Bengaluru, and stored at −20 °C for further analysis.

### 2.6. Testing of Samples

All serum samples were tested using an indigenous PPR competitive ELISA kit [11], which detects PPRV-specific antibodies against hemagglutinin (H) protein. The test measures the percentage inhibition (PI) of H protein-specific monoclonal antibodies binding to PPRV antigens. Samples with a PI of ≥40% were considered positive for presence of PPRV-specific antibodies [11].

### 2.7. Statistical Analysis

The seroprevalence of PPRV antibodies was calculated by dividing the number of positive animals by the total number tested. Univariate analysis was conducted to determine the association between seropositivity and various host factors (species, age, breed, and sex) using Pearson’s chi-squared test and Fisher’s exact test [12] using Microsoft Excel version 2016.

### 2.8. Vaccine Efficacy, Coverage and Effectiveness

The state employed the PPR Sungri 96 vaccine strain, produced at the Institute of Animal Health & Veterinary Biologicals, Hebbal, Bengaluru, Karnataka, through a state government initiative. This vaccine underwent rigorous quality testing independently by federal (central) authorities through third-party sampling and QC testing procedures. According to the WOAH/FAO guidelines, monitoring and evaluation are integral to a vaccination program, and an increase in the number of epi-units with a high number of protected age strata after post-vaccination is a positive indicator of a successful vaccination [4,7]. The terms “vaccine efficacy” and “vaccination effectiveness” are often used interchangeably but carry different meanings. The estimation of vaccine efficacy under field conditions, measured in terms of percentage seroconversion, relies on vaccinated animals demonstrating a sufficient and measurable protective immune response 28 days after receiving a single dose of the PPR vaccine. In contrast, vaccination effectiveness assesses how well populations are protected in the field by a vaccination program, taking into account both intrinsic and extrinsic factors such as vaccine storage, distribution, vaccination schedule, and vaccination coverages. Therefore, vaccine effectiveness in the population is an indirect estimate of the level of protection induced by the vaccine in the vaccinated goat and sheep population. It is calculated by multiplying the percentage of vaccination coverage [(number of animals vaccinated/total eligible animals for vaccination) × 100] in the target population by the percentage efficacy of the vaccine [4,7].

## 3. Results

**Figure 2:**
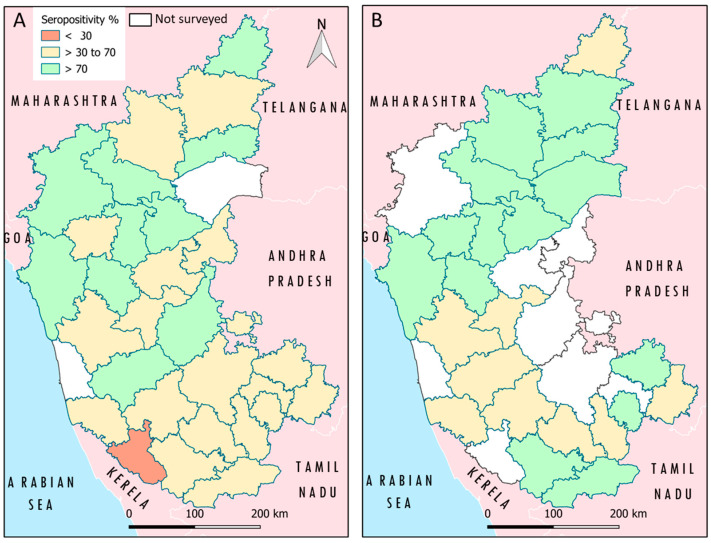
Distribution of PPRV antibody prevalence in small ruminants across various epidemiological units in different districts in Karnataka state, India. (**A**) Immunity status before implementation of mass vaccination (**B**) Post-vaccination immunity status. The districts shown in white indicate no sampling in that district.

**Figure 3:**
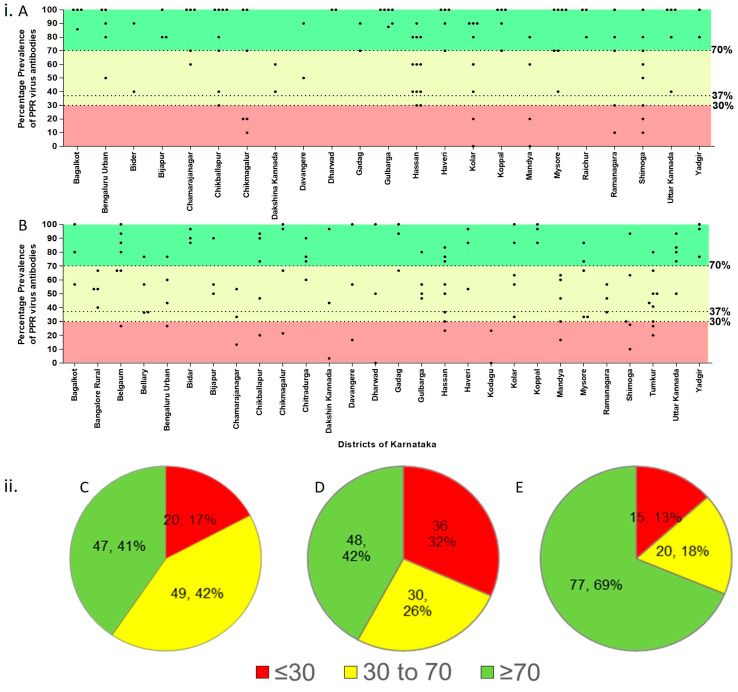
Distribution of epi-units based on percent positivity levels of PPRV antibodies in the studied state. (**i**) Frequency prevalence percentage of epi-units in different districts in Karnataka. (**A**) Prevalence/immunity status before implementation of mass vaccination, (**B**) Post-vaccination sero-monitoring. (**ii**) Distribution of epi-units based on percent positivity levels (**C**) Prevalence/immunity status before mass vaccination in all three age groups. (**D**) Prevalence/immunity status in the 6–12 months age group before vaccination (**E**) Post-vaccination sero-monitoring prevalence status in the 6–12 months age group.

The study demonstrated a 61.1% (CI 95%: 59–63) overall seroprevalence rate of PPRV antibodies in the population before the implementation of mass vaccination in small ruminants in the investigated epi-units of Karnataka state (Table S2). Specifically, sheep exhibited a seroprevalence of 64% (CI 95%: 61–67), while a 58% (CI 95%: 59–63) seroprevalence was observed in the goats. A detailed district-wise breakdown of the serum samples tested for PPRV antibodies before vaccination is presented in Table 1 and illustrated in Figure 2A. Regarding the immunity status in animals before the implementation of mass vaccination, a prevalence rate of 55.4% (687/1241), 62.4% (719/1153), and 66.2% (710/1072) was observed in the 6–12 months, 1–2 years, and over 2 years age groups, respectively. Chi-squared test results highlighted significant differences in the prevalence of PPRV antibodies between species (χ^2^ = 11.78, *p* < 0.05). Notably, both sheep (χ^2^ = 234.9, *p* < 0.05) and goats (χ^2^ = 253.5, *p* < 0.05) exhibited varied prevalences of PPRV antibodies across different districts (χ^2^ = 400.3, *p* < 0.05). Additionally, the analysis indicated significant associations (*p* < 0.05) between the presence of PPRV antibody and host factors such as breed (χ^2^ = 17.36, *p* < 0.05) and sex (χ^2^ = 48.71, *p* < 0.05) within the districts employed in this study. The distribution of PPRV antibody prevalence across various epi-units in different districts is depicted in Figure 3. Analysis of the data also revealed substantial disparities in the PPRV antibody prevalence among districts. Specifically, 41% of the epi-units (*n* = 47) had high prevalence rates exceeding 70%, while 17% of the epi-units (*n* = 20) had a seroprevalence below 30%, and 42% of the epi-units (*n* = 49) had prevalence rates between 30–70% before the implementation of mass vaccination. Further, in the 6–12 months age group, 42% of the epi-units (*n* = 48) had high prevalence rates (≥70%), while 32% of the epi-units (*n* = 36) had a seroprevalence below 30%, and 26% of the epi-units (*n* = 30) had prevalence rates between 30 and 70%.

The results of post-vaccination sero-monitoring revealed a promising seroconversion rate of 73.4% in the 6–12 months age group of small ruminants in various tested epi-units (Table S3) in the studied districts of Karnataka. A detailed district-wise breakdown of serum samples tested for PPRV antibodies after vaccination is presented in Table 2. The distribution of PPRV antibody prevalence in small ruminants across the districts is depicted in Figure 2B. Chi-squared analysis identified a non-significant association between the presence of PPRV antibody and the species, sex, and breed (*p* > 0.05), with better responders among nondescriptive animals of the small ruminants (Table 3) based on the available population samples tested. Furthermore, when evaluating the 6–12 months age group for vaccination immunity, over 69% of the epi-units (*n* = 77) achieved an immune response surpassing 70%, a marked improvement from 42% of the epi-units (*n* = 48) observed before vaccination. It complemented the post-vaccination response in the 6–12 months age group, indicating a 73.4% seroconversion. This translates to a vaccine efficacy rate of over 70% in the target sheep and goat populations. The study found an impressive overall vaccination achieving a 98.4% coverage during the study period in 23 districts in Karnataka. The subsequent vaccine effectiveness for the target populations was calculated to be >72%. Furthermore, only 13% of the epi-units had a prevalence rate of <37% antibodies which was significant and, presented a high risk of maintenance of the viruses in these epi-unit flocks.

## 4. Discussion

In India, national legislation plays a crucial role in addressing livestock diseases, supported by an integrated disease surveillance system. The prompt reporting of suspicious signs to local authorities reaches the District Officer, and upon laboratory confirmation, the Director of Animal Husbandry submits the monthly outbreak report to the Central Ministry through the Animal Disease Surveillance Report. Controlling PPR requires precise diagnosis, surveillance, and effective vaccination, involving legal frameworks, quarantine, movement restrictions, and intensive active surveillance. Underreporting of disease results from deficiencies in real-time field machinery due to an inadequate surveillance system. A state and national disease registry are vital for coordinated reporting, facilitating the efficient management of animal movements, early pathogen detection, and the implementation of biosecurity measures, as well as the immunization program at the eradication stage.

Karnataka, India, is at the forefront of mass vaccination for PPR eradication, aligning with the NSP. This study evaluated the prevalence of PPRV antibodies/immunity in sheep and goats through PVE surveys at the epi-unit level across Karnataka, following WOAH and FAO [7] guidelines. It highlighted the progress in PPR eradication, offering crucial insights into population immunity levels, vaccine efficacy, and vaccine effectiveness in real-world settings, and refining national strategies to achieve a PPR-free India. Stratified sampling design intentionally selected epi-units with substantial populations to ensure state representation and facilitate accurate prevalence estimation. The exclusion of epi-units with fewer than 500 populations did not significantly impact the estimation; despite not being selected, these units were not exempt from vaccination.

Karnataka, encompassing two of India’s 15 agro-climatic zones, initiated a “focused vaccination” approach in 2006, resulting in significant PPR control [6,13]. Since 2011–2012, aligned with the national PPR CP, the state has conducted mass vaccination campaigns, effectively keeping the disease under control [1,6,8]. Vaccination can be either a public or private initiative, contingent on a country’s stage, targeting either high-risk areas or the entire population [4]. India is in stage 2 of the GCES plan (as per the PPR Monitoring and Assessment Tools), and Karnataka state executed mass vaccination in 2023 following the NSP for PPR eradication. The state aimed to vaccinate >90% of the total sheep and goat population, excluding animals < 4 months old. At the beginning of January 2023, a total of 9.007 million doses of vaccines, subject to quality control, were centrally procured through DAHD, GoI, with the additional required doses (7.2 million) obtained subsequently. In 2023, Karnataka actively implemented the PPR Eradication Programme through intensive vaccination efforts. The state conducted mass vaccination campaigns in January 2023, covering more than 23 districts in the first month and subsequently extending coverage to the remaining districts. To assess PPRV antibody prevalence, the DAHD, GoI-approved indigenous PPR c-ELISA kit [11] was used. However, this kit could not differentiate between immunity from vaccination and natural infection since the Sungri 96 vaccine used in the vaccination program cannot differentiate infected from vaccinated animals (DIVA). Yet, prior surveys conducted during non-outbreak periods in states like Chhattisgarh reported the PPRV antibody prevalence above 50%, indicating successful mass vaccination [9,14]. Before mass vaccination in India, baseline seroprevalence rates ranged from 30 to 45% [14,15,16,17], providing a snapshot of the pre-vaccination prevalence status of the disease. However, in this study, the impact on the estimation of immunity status remained consistent, as only a few (three) sporadic outbreaks (one in each of the Bangalore Rural, Bellary, and Kalburgi districts) were reported during the period from April 2022 to March 2023. The notable (61%) seroprevalence of PPR suggests a resilient population immunity, attributed to the vaccination implemented under a controlled program since 2011 [1]. The sheep exhibited significantly higher seroprevalence than goats in both the pre- and post-vaccination periods. In India, goats are mainly reared for meat purposes while sheep are kept longer for wool production which may have contributed to the significantly higher seroprevalence in sheep than goats. Additionally, variations in seroprevalence may be influenced by differences in sample size, breed, management practices, and environmental factors like humidity or season [16]. However, significant differences and variations in the prevalence of PPRV antibodies between species in different districts may be attributed to the earlier implementation of the PPR vaccination program in the state; some districts were included in the vaccination program in previous years whereas some were not. In this study, 17% of the epi-units exhibited seroprevalence below 30% before vaccination, highlighting the necessity for intensified and targeted vaccination efforts in those districts.

The high turnover of small ruminants suggests that sustaining herd immunity above the 70% threshold requires consistently high vaccine coverage and regular vaccination campaigns within epi-units. Although vaccinating 100% of the target population is ideal, conventional expectations for PPR aim for a vaccination rate exceeding 70% population. Nevertheless, practical experiences such as Morocco’s PPR eradication efforts, demonstrated that achieving a 70% herd immunity was effective in preventing the spread of the virus [18]. Hence, generally the PVE methodology and the interpretation of results rely on a 70% immunity threshold at the epi-unit level. Evaluating vaccine effectiveness in small ruminants requires a multifaceted approach, such as estimating PPR incidence through outbreak reporting, participatory disease searches, and/or sero-epidemiological surveys [4,7]. In this study, a sero-epidemiological survey (post-vaccination evaluation) was carried out. The protocols for these serological surveys serve various purposes, ranging from establishing baseline immunity to evaluating the effectiveness of vaccination campaigns. To assess the latter, it is necessary to compare the proportion of epi-units with ≥70% of animals protected within the age stratum of 6–12 months to the baseline results [4,7]. According to the GCES guidelines of the WOAH/FAO, if a vaccine is effective, at least 50% of vaccinated epi-units should have a minimum of 70% seropositive animals [4,7]. In our findings, over 69% of these units met this criterion following vaccination, significantly higher than the 42% of the epi-units observed before vaccination. This indicates the efficacy of the PPR vaccine in preventing PPRV infection in the field. The observed 73.4% seroconversion at the field level indicates a high seropositivity of PPRV antibodies in vaccinated animals. Further, post-vaccination sero-monitoring showed that >87.3% of epi-units had a prevalence of >37% PPRV antibodies, indicating progress towards the eradication of PPR in the state. However, the prevalence of PPRV antibodies was below the desired baseline level of 30% prevalence in some of the epi-units (*n* = 15) which corroborates with the findings of Faris et al. [19]; they reported a post-vaccination seroconversion rate of 61.13%, indicating a relatively lower level of herd immunity when PPR vaccine, Nigeria 75/1 strain was used in sheep and goats in Ethiopia [19]. They attributed to the low seroconversion rate to the thermolabile nature of the vaccine. Moreover, the ELISA result in the study demonstrated a substantial percentage inhibition (PI) reaching 95%, signifying a robust positive reactivity of the serum in the test, similar to the positive control standard that comes with the kit. Researchers found a discrepancy between ELISA and VNT in detecting PPRV-positive antibodies, suggesting that even VNT-negative samples might show weak positivity (PI value 65–80) in ELISA [11,20] possibly because of the relatively higher sensitivity of ELISA than VNT.

Furthermore, Fournié, et al. [21], emphasized the importance of maintaining a minimum of 37% immune small ruminants in at least 71% of village populations in niche areas to prevent viral spread. In that study, only 13% of epi-units had a prevalence of <37% antibodies, with an overall prevalence rate of antibodies of 61%, indicating the prevention of viral spread in the studied state which explains why infrequent sporadic outbreaks were reported. Generally, these sporadic outbreaks were reported in niche areas, such as local markets, grazing fields where animals from various locations congregate, or through the introduction of new animals purchased from unknown sources to the existing flocks. The current analysis aims to assess prevalence estimation, considering the state as the final entity to inform the impact of vaccination rather than focusing on individual districts. Among the districts, the highest observed prevalence of antibodies was in Koppal, followed by Gadag, Bidar, Yadgir, Bagalkot, Haveri, Uttar Kannada, etc., while Kodagu district exhibited the lowest prevalence. Similarly, the highest observed post-vaccination prevalence of antibodies was in the Dharwad district, followed by Bagalkot, Gulbarga, Raichur, Koppal, Haveri, etc., with the lowest immune response observed in the Mandya district. These variations may have arisen from epi-units within the state being randomly selected, acknowledging that the units chosen for the pre-vaccination survey may differ from the ones selected for the post-vaccination study. According to the records of the state animal husbandry department, only four outbreaks occurred (in Kolar and Bellary districts) in the financial year from April 2021 to March 2022, and three outbreaks were recorded (in Bangalore Rural, Bellary, Kalburgi districts) in 2022–2023.

This study provides significant insights into the effectiveness of the vaccine in real-world settings, considering its distribution, administration, and the population. Even though Karnataka carried out a mass vaccination campaign, the non-achievement of optimal herd/population immunity highlights the challenges faced which may be due to administrative reasons especially the non-availability of vaccines on time. This study recommends adopting an annual mass vaccination program over three to four consecutive years under the PPR-EP initiative to achieve the desired level of herd immunity at the cluster/village level for the eradication of the disease in Karnataka. In conclusion, this study presents the first report on PPR post-vaccination sero-monitoring, vaccine efficacy, and vaccine effectiveness in the region, which had implemented mass vaccination under PPR-EP. In order to enhance eradication efforts and achieve the desired PPRV antibody prevalence, it is crucial to intensify mass vaccination campaigns in areas that have not reached the desired herd immunity level. The surveys conducted in this study have unveiled the prevalence rate of PPRV antibodies in sheep and goats, providing an accurate representation of the target population in Karnataka, India. This study also offers valuable insights for developing effective eradication strategies against PPR, especially in the context of vaccination efforts in Karnataka which can be extended to other Indian states.

## 5. Conclusions and Perspectives

The prevalence of antibodies varied among districts where the program had been implemented due to disparities in vaccination efforts. This underscores the need for comprehensive vaccination campaigns and active surveillance to establish PPR-free zones. To expedite the eradication of PPR, this study recommends conducting mass vaccination campaigns with a target of achieving over 95% coverage for animals over four months of age. The goal is to attain more than 85% seroconversion, thereby achieving at least >70% herd immunity through vaccination. Consequently, annual mass vaccinations over three to four consequent years are proposed under the PPR-EP of India to achieve the desired level of immunity at the cluster level.

In future, a third PVE survey will be conducted within 60–90 days after the second PPR mass vaccination during the year 2024, followed by subsequent annual vaccinations to monitor the trend of population immunity over time. This approach not only measures susceptibility levels in the vaccinated population but also assesses the effectiveness of the vaccination campaign, compares results within different age groups, and observes the trend of increasing epi-units with over 70% of animals protected. Once desired cluster-level prevalence is achieved with minimal or sporadic outbreaks, efforts can be redirected towards targeted areas such as bordering districts (creating buffer zones with neighboring states), animal markets, and checkpoints to prevent the introduction of PPR. Therefore, a comprehensive strategy involving mass vaccination, monitoring, and surveillance should be extended to various states and zones of the country. Additionally, along with continuous monitoring, zoning PPR risk regions and implementing mass vaccination programs are of utmost importance. When India is ready to declare provisional freedom from PPR, surveillance must adhere to the WOAH/FAO guidelines to confirm this status in unvaccinated sheep and goats.

## Figures and Tables

**Table 1:** Details of the PPRV antibodies prevalence in small ruminants in Karnataka before implementation of mass vaccination. CI—confidence interval.

Name of the District	No. of Village/Epi-Units	No. of Taluks in the Districts	No. of Serum Samples Screened	No. of Samples Positive in ELISA	Prevalence (%)	No. of Serum Samples Screened	No. of Samples Positive in ELISA	Prevalence (%)	No. of Serum Samples Screened	No. of Samples Positive in ELISA	Prevalence (%)
			Sheep			Goat			Total		
Bagalkot	3	2	54	45	83.3	36	26	72.2	90	71	78.9
Bangalore Rural	4	2	75	46	61.3	45	18	40.0	120	64	53.3
Belgaum	7	7	67	59	88.1	143	97	67.8	210	156	74.3
Bellary	4	3	81	43	53.1	31	16	51.6	112	59	52.7
Bengaluru Urban	4	3	51	24	47.1	69	38	55.1	120	62	51.7
Bidar	3	2	30	26	86.7	60	56	93.3	90	82	91.1
Bijapur	3	3	49	29	59.2	41	30	73.2	90	59	65.6
Chamarajanagar	3	2	56	21	37.5	34	09	26.5	90	30	33.3
Chikballapur	5	4	114	74	64.9	36	23	63.9	150	97	64.7
Chikmagalur	4	1	103	72	69.9	15	13	86.7	118	85	72.0
Chitradurga	4	3	93	70	75.3	27	20	74.1	120	90	75.0
Dakshin Kannada	3	2	-	-	-	90	43	47.8	90	43	47.8
Davangere	3	2	62	30	48.4	28	22	78.6	90	52	57.8
Dharwad	3	3	28	24	85.7	62	21	33.9	90	45	50.0
Gadag	3	3	45	43	95.6	45	35	77.8	90	78	86.7
Gulbarga	4	2	18	14	77.8	102	56	54.9	120	70	58.3
Hassan	8	4	163	93	57.1	77	36	46.8	240	129	53.8
Haveri	3	3	42	37	88.1	48	34	70.8	90	71	78.9
Kodagu	2	2	15	0	0	45	07	15.6	60	07	11.7
Kolar	5	3	129	89	69	21	13	61.9	150	102	68.0
Koppal	3	1	72	67	93.1	18	18	100	90	85	94.4
Mandya	5	4	78	42	53.8	72	23	31.9	150	65	43.3
Mysore	5	3	66	40	60.6	84	48	57.1	150	88	58.7
Ramanagara	3	3	33	16	48.5	57	26	45.6	90	42	46.7
Shimoga	5	4	40	15	37.5	109	52	47.7	149	67	45.0
Tumkur	9	7	197	99	50.3	70	22	31.4	267	121	45.3
Uttar Kannada	5	2	42	20	47.6	108	94	87.0	150	114	76.0
Yadgir	3	2	45	45	100	45	37	82.2	90	82	91.1
Grand Total	116	82	1848	1183	64	1618	933	57.7	3466	2116	61.1
Chi-squared value; *p*-value and Prevalence at 95% CI			χ^2^: 234.86, *p* < 0.05		CI 95%: 61 to 67	χ^2^: 253.5, *p* <0.05		CI 95%: 55 to 61	χ^2^: 400.34, *p* < 0.05		CI 95%: 59 to 63

**Table 2:** Details of the post-vaccination PPRV antibodies prevalence in small ruminants in Karnataka. CI—confidence interval.

Name of the Districts in Karnataka	No. of Village/Epi-Units	No. of Taluks in the Districts	No. of Serum Samples Screened	No. of Samples Positive	Prevalence (%)	No. of Serum Samples Screened	No. of Samples Positive	Prevalence (%)	No. of Serum Samples Screened	No. of Samples Positive	Prevalence (%)
			Sheep			Goat			Total		
Bagalkot	4	2	31	30	96.8	06	06	100	37	36	97.3
Bengaluru Urban	5	3	28	24	85.7	22	18	81.8	50	42	84.0
Bider	2	1	06	02	33.3	14	11	78.6	20	13	65.0
Bijapur	3	2	13	10	76.9	17	16	94.1	30	26	86.7
Chamarajanagar	5	1	33	30	90.9	17	13	76.5	50	43	86.0
Chikballapur	8	4	67	51	76.1	12	10	83.3	79	61	77.2
Chikmagalur	6	3	45	24	53.3	14	07	50.0	59	31	52.5
Dakshina Kannada	2	1	-	-	-	20	10	50.0	20	10	50.0
Davangere	2	2	18	12	66.7	02	02	100	20	14	70.0
Dharwad	2	1	11	11	100	09	09	100	20	20	100
Gadag	2	2	15	12	80.0	05	04	80.0	20	16	80.0
Gulbarga	6	6	07	06	85.7	51	50	98.0	58	56	96.6
Hassan	12	5	73	42	57.5	47	27	57.4	120	69	57.5
Haveri	5	3	34	30	88.2	16	16	100	50	46	92.0
Kolar	8	5	63	38	60.3	16	09	56.3	79	47	59.5
Koppal	5	3	45	41	91.1	05	05	100	50	46	92.0
Mandya	4	3	19	09	47.4	21	07	33.3	40	16	40.0
Mysore	7	4	30	23	76.7	40	35	87.5	70	58	82.9
Raichur	3	3	30	28	93.3	-	-	-	30	28	93.3
Ramanagara	5	3	30	17	56.7	20	12	60.0	50	29	58.0
Shimoga	8	3	46	20	43.5	34	22	64.7	80	42	52.5
Uttar Kannada	5	2	11	09	81.8	39	33	84.6	50	42	84.0
Yadgir	2	2	16	14	87.5	04	04	100	20	18	90.0
Grand Total	111	64	671	483	72.0	431	326	75.6	1102	809	73.4
Chi-squared value; *p*-value and Prevalence at (CI) 95% CI			χ^2^: 98.22, *p* < 0.05		CI 95%: 68 to 76	χ^2^: 85.37, *p* < 0.05		CI 95%: 71 to 80	χ^2^: 167.24, *p* < 0.05		CI 95%: 70 to 76

**Table 3:** Chi-squared analysis of results of PPRV antibody prevalence in small ruminants in Karnataka.

	Before Implementation of Mass Vaccination					Post-Vaccination Sero-Monitoring				
Variables	No. of Serum Samples Screened	No. of Samples Positive	Prevalence (%)	χ^2^	*p-*Value	No. of Serum Samples Screened	No. of Samples Positive	Prevalence (%)	χ^2^	*p-*Value
**Sheep**	1900	1209	63.6	11.78	<0.05 *	671	483	72.0	1.79	>0.05
**Goat**	1566	907	57.9			431	326	75.6		
**Male**	716	356	49.7	48.71	<0.05 *	304	214	70.4	1.95	>0.05
**Female**	2750	1760	64.0			798	595	74.6		
**6–12 months**	1163	640	55.0	30.54	>0.05	1102	809	73.4	-	-
**1–2 Years**	1231	766	62.2			-	-	-		
**Above 2 Years**	1072	710	66.2			-	-	-		
**Non-descriptive**	3368	2076	61.6	17.36	<0.05 *	1059	779	73.6	0.30	>0.05
**Crossbreed**	98	40	40.8			43	30	69.8		

* Significance at 95% confidence level.

## Data Availability

The data presented in this study are available in the manuscript and in the Supplementary Files. All datasets supporting our findings are available at the PPR Research Laboratory of the ICAR-NIVEDI, Bengaluru on reasonable request from the corresponding author.

## Supplementary Materials

The following supporting information can be downloaded at: https://www.mdpi.com/article/10.3390/v16030333/s1, Table S1: Sampling plan for the comprehensive Post Vaccination Evaluation strategic survey Protocol I approach as per GCES guidelines of WOAH/FAO. Table S2: Details of the PPRV antibodies prevalence/immune status in small ruminants in different epi-units of various districts in Karnataka before the implementation of mass vaccination. Table S3: Details of the post-vaccination PPRV antibodies prevalence in small ruminants in different epi-units in various districts of Karnataka.
