# Supplementary material for: Post-Vaccination Sero-Monitoring of Peste des Petits Ruminants in Sheep and Goats in Karnataka: Progress towards PPR Eradication in India
Source: Viruses. 2024 Feb 22;16(3):333. doi: 10.3390/v16030333 (PMC10974862; doi:10.3390/v16030333)
Supplement: Supplementary file 1 [file viruses-16-00333-s001.zip › viruses-2835843-supplementary.pdf]

<u>**Supplementary Documents**</u>

Title: Seromonitoring of Peste des Petits Ruminants in Sheep and Goats in Karnataka: Progress Towards PPR Eradication in India

Table S1: Sampling plan for the comprehensive Post Vaccination Evaluation (PVE) strategic survey Protocol I approach as per GCES guidelines of WOAH/FAO

Country	India	Remarks and Samples size estimation
State	Karnataka	The study area is purposefully selected as the state
Villages /Epiunits	Random selection of 108-120 villages in the study region	Villages as an epidemiological unit (epi-unit) are to be selected randomly concerning the size of the population. Before the stratified randomization of villages, the trimming of the sampling frame (source of villages from which samples are to be drawn) was employed for 500 animals per village as an inclusion criterion to have a substantial population
Household or Flocks	3 or 4 flocks in the village by random/ convenient selection	Based on the small ruminant population, a maximum of 3-4 animals per each eligible age group or stratum (age = between 6-12 months, one to 2 years and > 2 years) or 3 animals in 6-12 months age group and 6 animals in more than one-year age group were selected conveniently.
Animal units for Pre-vaccination Survey (Present Study- First Survey)	Pre-vaccination (Baseline seroprevalence before implementation of vaccination)	Total No. of samples to be tested for before implementation of mass vaccination.<br>Minimum animal samples size = 108 x 27 = 2916 samples or Maximum animal samples size = 108 x 30= 3240 samples<br>Pre-vaccination -Different age groups categories – 30 samples (10 samples from 6-12 months; 10 from > 1-2 years and 10 from > 2 years aged animals).
Animal Units for Post-vaccination Survey (Present Study- Second Survey)	Post-vaccination immune response	Total No. of samples to be tested after implementation of mass vaccination<br>Minimum animal samples size = 108 x 9 = 972 samples or Maximum animal samples size = 108 x 10= 1080 samples.<br>Post-vaccination- Vaccine efficacy/Sero conversion- one age group– 10 samples (10 samples from 6-12 months aged animals).
Animal units for Population Immunity Survey<br><br>(Future Study- Third Survey)	Population immunity trends- each year after vaccination	Total No. of samples to be tested for population immunity after mass vaccination each year.<br>Minimum animal samples size = 108 x 27 = 2916 samples or Maximum animal samples size = 108 x 30= 3240 samples<br>Population Immunity: Different age groups categories – 30 samples (10 samples from 6-12 months; 10 from > 1-2 years and 10 from > 2 years aged animals).

Table S2: Details of the PPRV antibodies prevalence /immune status in small ruminants in different epidemiological units of various districts in Karnataka before the implementation of mass vaccination

Name of Village (Epi-Units)	Name of Taluk/ Block	Name of District	No. of serum samples screened	No. of samples positive in ELISA	Prevalence of PPRV antibodies status %
Siraguppi	Bagalkot	Bagalkot	30	17	56.7
Timmapur	Bagalkot	Bagalkot	30	30	100
Junnur	Mudhol	Bagalkot	30	24	80.0
Bhumenahalli	Dod Ballapur	Bangalore Rural	30	20	66.7
Peramagondanahalli	Dod Ballapur	Bangalore Rural	30	16	53.3
Tiruvanahalli (D)	Dod Ballapur	Bangalore Rural	30	12	40.0
Ganagalu	Hosakote	Bangalore Rural	30	16	53.3
Kodni	Chikodi	Belgaum	30	24	80.0
Yadwad	Gokak	Belgaum	30	08	26.7
Hulloli	Hukeri	Belgaum	30	30	100
Naganur-Ward No.5	Naganur	Belgaum	30	20	66.7
Bhendwad	Raybag	Belgaum	30	26	86.7
Wakkund	Sampgaon	Belgaum	30	20	66.7
Sankeshwar (TMC) -	Sankeshwar	Belgaum	30	28	93.3
Rupanagudi	Bellary	Bellary	30	11	36.7
Vaddahatti	Bellary	Bellary	30	17	56.7
Alabur	Hagaribommanahalli	Bellary	22	08	36.4
Kudureduvu	Kudligi	Bellary	30	23	76.7
Adigarakallahalli	Anekal	Bengaluru Urban	30	18	60.0
Yerappanahalli	Bangalore East	Bengaluru Urban	30	23	76.7
Bangalore (M Corp.)	Bangalore North	Bengaluru Urban	30	08	26.7
Kukkanahalli	Bangalore North	Bengaluru Urban	30	13	43.3
Alur(K)	Aurad	Bidar	30	27	90.0
Balur	Bhalki	Bidar	30	26	86.7
Yenkura(X)	Bhalki	Bidar	30	29	96.7
Atharga	Indi	Bijapur	30	27	90.0
Bhantnur	Muddebihal	Bijapur	30	17	56.7
Nalatawad -Ward	Nalatawad	Bijapur	30	15	50.0
Kiragasur	Chamarajanagar	Chamarajanagar	30	10	33.3
Ajjipura	Kollegal	Chamarajanagar	30	16	53.3
Thotadmanegalu	Kollegal	Chamarajanagar	30	04	13.3
Ooduvaripalli	Bagepalli	Chikballapur	30	27	90.0
Kuthappanahalli	Chintamani	Chikballapur	30	06	20.0
Thammepalli	Chintamani	Chikballapur	30	22	73.3
Maralur	Gauribidanur	Chikballapur	30	28	93.3
Lakkenahalli (34)	Sidlaghatta	Chikballapur	30	14	46.7
Chatnahalli	Kadur	Chikmagalur	30	20	66.7
Chowlihiriyur	Kadur	Chikmagalur	30	29	96.7
Hiregarje	Kadur	Chikmagalur	28	06	21.4

Name of Village (Epi-Units)	Name of Taluk/ Block	Name of District	No. of serum samples screened	No. of samples positive in ELISA	Prevalence of PPRV antibodies status %
Shettyhalli	Kadur	Chikmagalur	30	30	100
Nannivala	Challakere	Chitradurga	30	27	90.0
Hunesekatte	Chitradurga	Chitradurga	30	23	76.7
Thurebailu	Chitradurga	Chitradurga	30	18	60.0
Venkatapura	Molakalmuru	Chitradurga	30	22	73.3
Ervuailu	Mangalore	Dakshin Kannada	30	29	96.7
Talapady (Ct)	Mangalore	Dakshin Kannada	30	13	43.3
Ullal (TP) -Ward	Ullal	Dakshin Kannada	30	01	3.30
Kereyagalahalli	Davanagere	Davangere	30	30	100
Malagondanahalli	Davanagere	Davangere	30	05	16.7
Towdur	Harapanahalli	Davangere	30	17	56.7
Channapur	Hubli	Dharwad	30	15	50.0
Bilebal	Kundgol	Dharwad	30	00	00.0
Gummagol	Navalgund	Dharwad	30	30	100
Vithalapur	Mundargi	Gadag	30	28	93.3
Naregal (TP) -Ward	Naregal	Gadag	30	20	66.7
Hullur	Shirhatti	Gadag	30	30	100
Khandal	Gulbarga	Gulbarga	30	17	56.7
Malsapur	Gulbarga	Gulbarga	30	15	50.0
Kalhangerga	Jevargi	Gulbarga	30	14	46.7
Magengera	Jevargi	Gulbarga	30	24	80.0
Bevinahalli	Arsikere	Hassan	30	17	56.7
Byalekere	Arsikere	Hassan	30	15	50.0
Guttinakere	Arsikere	Hassan	30	09	30.0
Kenkere	Arsikere	Hassan	30	25	83.3
Hirihalli	Belur	Hassan	30	23	76.7
Shanthigrama	Hassan	Hassan	30	11	36.7
Anekannambadi	HoleNarsipur	Hassan	30	22	73.3
Kabbur	HoleNarsipur	Hassan	30	07	23.3
Janginakoppa	Hangal	Haveri	30	16	53.3
Havanur	Haveri	Haveri	30	26	86.7
Ukkund	Ranibennur	Haveri	30	29	96.7
Kudumangalore	Somvarpet	Kodagu	30	07	23.3
Nokya	Virajpet	Kodagu	30	00	00.0
Kadarinatha	Bangarapet	Kolar	30	17	56.7
Lakkenahalli (74)	Bangarapet	Kolar	30	10	33.3
Upasapura	Bangarapet	Kolar	30	30	100
Nachahalli	Mulbagal	Kolar	30	19	63.3
Doddamaladoddi	Srinivaspur	Kolar	30	26	86.7
Donnegudda	Kushtagi	Koppal	30	29	96.7
Hiremannapur	Kushtagi	Koppal	30	30	100
Topalkatti	Kushtagi	Koppal	30	26	86.7
Billenahalli	Krishnarajpet	Mandya	30	18	60.0
Maraliga	Maddur	Mandya	30	09	30.0

Name of Village (Epi-Units)	Name of Taluk/ Block	Name of District	No. of serum samples screened	No. of samples positive in ELISA	Prevalence of PPRV antibodies status %
Marasinganahalli	Maddur	Mandya	30	05	16.7
Cheeranahalli	Mandya	Mandya	30	14	46.7
Shivanahalli	Nagamangala	Mandya	30	19	63.3
Hanchya	Mysore	Mysore	30	10	33.3
Mavinahalli	Mysore	Mysore	30	10	33.3
Belagunda	Nanjangud	Mysore	30	26	86.7
Kallipura	Tirumakudal - Narsipur	Mysore	30	22	73.3
T. Bettahalli	Tirumakudal - Narsipur	Mysore	30	20	66.7
Brahmanipura	Channapatna	Ramanagara	30	17	56.7
Mahalli	Kanakapura	Ramanagara	30	14	46.7
Ibbalakahalli	Ramanagara	Ramanagara	30	11	36.7
Balemaranahalli	Bhadravati	Shimoga	29	08	27.6
Belagavi	Shikaripura	Shimoga	30	28	93.3
Kadenandihalli	Shikaripura	Shimoga	30	19	63.3
Shimoga (CMC) -	Shivamogga	Shimoga	30	03	10.0
Chikkadagodu	Sorab	Shimoga	30	09	30.0
Bangaragere	Chiknayakanhalli	Tumkur	30	06	20.0
Byadagere	Gubbi	Tumkur	30	15	50.0
Bukkapatna	Koratagere	Tumkur	27	11	40.7
Muddenahalli	Madhugiri	Tumkur	30	13	43.3
Muddaganahalli	Pavagada	Tumkur	30	15	50.0
Karehalli	Sira	Tumkur	30	09	30.0
Tadakalur	Sira	Tumkur	30	24	80.0
Kunikenahalli	Turuvekere	Tumkur	30	08	26.7
Mallur	Turuvekere	Tumkur	30	20	66.7
Ghadiyal	Haliyal	Uttar Kannada	30	24	80.0
Jatga	Haliyal	Uttar Kannada	30	25	83.3
Hungunda	Mundgod	Uttar Kannada	30	28	93.3
Indoor	Mundgod	Uttar Kannada	30	15	50.0
Mainalli	Mundgod	Uttar Kannada	30	22	73.3
Bholkadamgera	Shahpur	Yadgir	30	30	100
Karkihalli	Shahpur	Yadgir	30	29	96.7
Shorapur (TMC) -	Shorapur	Yadgir	30	23	76.7
Grand Total (116)	82	28	3466	2116	61.1 % (CI 95%: 59 to 63)

Table S3: Details of the post-vaccination PPRV antibodies prevalence in small ruminants in different epidemiological units in various districts of Karnataka

Name of Village (Epi-Units)	Name of Taluk/ Block	Name of District	No. of serum samples screened	No. of samples positive in ELISA	Prevalence of PPRV antibodies status %
Fakeerabudihal	Badami	Bagalkot	10	10	100
Dammur	Hungund	Bagalkot	10	10	100
Ilal	Hungund	Bagalkot	10	10	100
Virapur	Hungund	Bagalkot	07	06	85.7
Chikkanayakanahalli	Bangalore East	Bengaluru Urban	10	05	50.0
Mandur	Bangalore East	Bengaluru Urban	10	10	100
Ward no.50 (benniganahalli)	Bangalore East	Bengaluru Urban	10	09	90.0
Guddadahalli	Bangalore North	Bengaluru Urban	10	08	80.0
Doddamaranahalli	Bangalore South	Bengaluru Urban	10	10	100
Ghotala	Basavakalyan	Bider	10	04	40.0
Rajeshwar	Basavakalyan	Bider	10	09	90.0
Mulawad	Basavana Bagevadi	Bijapur	10	08	80.0
Dyaberi	Bijapur	Bijapur	10	08	80.0
Malakandevarahatti	Bijapur	Bijapur	10	10	100
Baalagunase	Kollegal	Chamarajanagar	10	10	100
Gaajanooru	Kollegal	Chamarajanagar	10	07	70.0
Kunagalli	Kollegal	Chamarajanagar	10	10	100
Settahalli	Kollegal	Chamarajanagar	10	10	100
Tellanur	Kollegal	Chamarajanagar	10	06	60.0
Nallaguttapalli	Bagepalli	Chikballapur	10	10	100
Thimmampalli	Bagepalli	Chikballapur	10	10	100
Yellampalli	Bagepalli	Chikballapur	09	09	100
Manchanabele	Chikkaballapura	Chikballapur	10	03	30.0
Marappanahalli	Chikkaballapura	Chikballapur	10	10	100
Yalagalahalli	Chikkaballapura	Chikballapur	10	07	70.0
Kuruburu	Chintamani	Chikballapur	10	08	80.0
Vemagallu	Sidlaghatta	Chikballapur	10	04	40.0
Devagondanahalli	Chikmagalur	Chikmagalur	10	07	70.0
Sindigere	Chikmagalur	Chikmagalur	10	02	20.0
B.T. Mallenahalli	Kadur	Chikmagalur	10	10	100
Devanur	Kadur	Chikmagalur	09	09	100
P. Kodihalli	Kadur	Chikmagalur	10	01	10.0
Gadihalli	Tarikere	Chikmagalur	10	02	20.0
Aithoor	Puttur	Dakshina Kannada	10	06	60.0
Kadaba	Puttur	Dakshina Kannada	10	04	40.0
Nagarasanahalli	Davanagere	Davangere	10	09	90.0
Mallapura	Jagalur	Davangere	10	05	50.0
Harobelavadi	Dharwad	Dharwad	10	10	100
Hebballi	Dharwad	Dharwad	10	10	100
Guddadbudihal	Mundargi	Gadag	10	07	70.0

Asuti	Ron	Gadag	10	09	90.0
D. Ghangapur	Afzalpur	Gulbarga	10	10	100
Madan Hipperga	Aland	Gulbarga	10	10	100
Yetabarpur	Chincholi	Gulbarga	10	10	100
Hongunta	Chitapur	Gulbarga	08	07	87.5
Ganjalnkhed	Gulbarga	Gulbarga	10	10	100
Medak	Sedam	Gulbarga	10	09	90.0
Hullangala	Arkalgud	Hassan	10	09	90.0
Belagumba	Arsikere	Hassan	10	03	30.0
Chikkalkur	Arsikere	Hassan	10	04	40.0
Hullekere	Arsikere	Hassan	10	08	80.0
Mududi	Arsikere	Hassan	10	03	30.0
Nerlige	Arsikere	Hassan	10	08	80.0
Paduvanahalli	Arsikere	Hassan	10	08	80.0
Rampura	Arsikere	Hassan	10	04	40.0
Thirupathihalli	Arsikere	Hassan	10	06	60.0
Rajanasiriyur	Belur	Hassan	10	04	40.0
Valagerehalli	Channarayapatna	Hassan	10	06	60.0
S. Ankanahalli	Hole Narsipur	Hassan	10	06	60.0
Chattra	Byadgi	Haveri	10	10	100
Kalledevar	Byadgi	Haveri	10	10	100
Hirelingadahalli	Haveri	Haveri	10	10	100
Choudayya danapur	Ranibennur	Haveri	10	09	90.0
Itagi	Ranibennur	Haveri	10	07	70.0
Thalur	Bangarapet	Kolar	10	04	40.0
Maliyappanahalli	Kolar	Kolar	10	06	60.0
Kuppur	Malur	Kolar	10	08	80.0
Nutuve	Malur	Kolar	10	09	90.0
Byatanur	Mulbagal	Kolar	10	02	20.0
Nachagundlahalli	Mulbagal	Kolar	10	09	90.0
Sangasandra	Mulbagal	Kolar	10	09	90.0
Kornalli	Srinivaspur	Kolar	09	-	-
Indaragi	Koppal	Koppal	10	10	100
Sangapur	Koppal	Koppal	10	09	90.0
Hiremannapur	Kushtagi	Koppal	10	10	100
Topalakatti	Kushtagi	Koppal	10	10	100
Chikmyageri	Yelbarga	Koppal	10	07	70.0
Belathur	Maddur	Mandya	10	-	-
Chottananhalli	Malavalli	Mandya	10	08	80.0
Ravani	Malavalli	Mandya	10	02	20.0
Anunahalli	Pandavapura	Mandya	10	06	60.0
Manuganahalli	Hunsur	Mysore	10	04	40.0
Moduru	Hunsur	Mysore	10	07	70.0
Hadajana	Mysore	Mysore	10	10	100
Huyilalu	Mysore	Mysore	10	10	100
Udburu	Mysore	Mysore	10	07	70.0

Nerale	Nanjangud	Mysore	10	10	100
Somanathapura	Tirumakudal - Narsipur	Mysore	10	10	100
Mincheri	Lingasugur	Raichur	10	10	100
Pamanakalluru	Maski	Raichur	10	08	80.0
Polkamdoddi	Raichur	Raichur	10	10	100
Chakkere	Channapatna	Ramanagara	10	08	80.0
Thimmasandra	Channapatna	Ramanagara	10	07	70.0
Hunsanahalli	Kanakapura	Ramanagara	10	01	10.0
Shuivanegowdanadoddi	Kanakapura	Ramanagara	10	03	30.0
Bilagumba	Ramanagara	Ramanagara	10	10	100
Guledahalli	Shikaripura	Shimoga	10	08	80.0
Kaginalli	Shikaripura	Shimoga	10	03	30.0
Kappanahalli	Shikaripura	Shimoga	10	01	10.0
Kattigehalla	Shikaripura	Shimoga	10	10	100
Thumrihosuru	Shikaripura	Shimoga	10	06	60.0
Agasavalli hosuru	Shimoga	Shimoga	10	05	50.0
Gejjenahalli	Shimoga	Shimoga	10	02	20.0
Anavatti	Sorab	Shimoga	10	07	70.0
Gadiyal	Haliyal	Uttar Kannada	10	04	40.0
Jatga	Haliyal	Uttar Kannada	10	08	80.0
Hungunda	Mundgod	Uttar Kannada	10	10	100
Indoor	Mundgod	Uttar Kannada	10	10	100
Mainalli	Mundgod	Uttar Kannada	10	10	100
Kekkera (tp) - ward no.14	Kekkera	Yadgir	10	08	80.0
Kadechur	Yadgir	Yadgir	10	10	100
Grand total (111)	64	23	1102	809	73.4% CI 95%: 70 to 76
